# Perinatal outcome in monoamniotic twin pregnancies during a 10-year period: a single center, descriptive study

**DOI:** 10.1007/s00404-022-06506-3

**Published:** 2022-03-26

**Authors:** Ingrid Söderhult, Eleonor Tiblad, Lotta Herling

**Affiliations:** 1 Department of Obstetrics and Gynecology, Center for Fetal Medicine, Karolinska University Hospital, Stockholm, Sweden; 2 Clinical Epidemiology Division, Department of Medicine Solna, Karolinska Institutet, Stockholm, Sweden; 3 Department of Clinical Science, Intervention and Technology-CLINTEC, Karolinska Institutet, Stockholm, Sweden

**Keywords:** Monoamniotic twin pregnancy, Perinatal survival, Pregnancy complication, Pregnancy outcome, Twin pregnancy

## Abstract

**Purpose:**

To retrospectively investigate perinatal outcome of monoamniotic twin pregnancies in a tertiary center during a 10 year period.

**Methods:**

A retrospective analysis of all monoamniotic pregnancies managed at Karolinska University Hospital, Stockholm, Sweden 2010–2019 was performed. The primary outcomes were live birth rate, neonatal death and perinatal survival. The secondary outcomes were late miscarriage, gestational age at delivery and frequency of fetal complications.

**Results:**

Twenty-two monoamniotic pregnancies, with 44 fetuses, were identified. Thirty-five of 44 fetuses (80%) were liveborn. Of 36 fetuses reaching 24 weeks gestation, 35 (97%) were liveborn. There were no neonatal deaths, thus the perinatal survival was 97%. The mean gestational age at birth was 32.5 weeks (SD ± 1.5).

**Conclusions:**

The live birth rate and perinatal survival of monoamniotic pregnancies managed at Karolinska University Hospital was high and comparable to previously published data.

## Introduction

Monochorionic monoamniotic (MCMA) twin pregnancies have the highest perinatal mortality rate of all twins. In monochorionic diamniotic (MCDA) and dichorionic diamniotic (DCDA) twin pregnancies, perinatal mortality rates of approximately 10% and 2%, respectively, have been reported [1, 2]. The perinatal mortality rate in MCMA twins has previously been reported to be 30–70% [3]. With more intensive fetal surveillance, perinatal mortality has declined to 8–11% after 24–26 weeks gestation [4, 5]. The rate of intrauterine fetal demise (IUFD) has declined to 6–8% after 24 and 26 weeks gestation [4, 5]. Although there has been progress in managing these pregnancies, the mortality rate is still high and varies between studies [2, 6].

Twin–twin transfusion syndrome (TTTS), selective intrauterine growth restriction (sIUGR) and IUFD are complications that can appear in MCMA pregnancies due to unequal placental sharing and/or hemodynamic imbalance between the fetuses caused by vascular anastomoses in the placenta connecting the fetal circulations [5–7]. The anastomoses are also a cause of the increased risk of sudden fetal demise in MCMA twins as well as of the increased risk of double intrauterine fetal demise (dIUFD) after loss of one fetus [7]. Except the complications due to vascular anastomoses, MCMA twins also have an increased risk of congenital anomalies, umbilical cord complications and iatrogenic and spontaneous preterm delivery [6, 8, 9].

Management of monoamniotic twin pregnancies varies and the optimal timing for elective delivery, which often takes place at 32–34 weeks gestation, is yet unknown [6, 9]. In some countries inpatient management in the third trimester is routine, while in Sweden almost all cases are managed as outpatients [4]. A multinational cohort study by Saccone et al*.* evaluated the perinatal mortality rate for patients managed as inpatients versus outpatients in uncomplicated monoamniotic twin pregnancies. The results suggested that perinatal mortality rate was lower in the inpatient group but the results were inconclusive [4].

Pregnancy and perinatal outcome of MCMA twins in Sweden is not known. There is a lack of evidence-based guidelines and management is often based on expert opinions [5, 6, 10]. The aim of this study was to retrospectively investigate perinatal outcome of MCMA twin pregnancies in a tertiary center during a 10 year period.

## Materials and methods

All MCMA pregnancies managed at Karolinska University Hospital, Stockholm, Sweden 2010–2019 were included. The hospital serves as referral center for complicated multiple pregnancies in the region of Stockholm. Exclusion criteria were cases with chromosomal abnormalities, conjoined twins, cases complicated by twin reversed arterial perfusion (TRAP) sequence, multiple pregnancies with more than two fetuses, cases referred from outside the Stockholm region for fetal therapy and cases that first were followed as monoamniotic but later diagnosed as diamniotic by the placental pathology examination. MCMA pregnancies were identified and data extracted from the medical record system using ICD-10 codes (O30.0 before 2011 and O30.0C from 2011). Data obtained regarding maternal demographics were age, parity, body mass index (BMI), pregnancy type, gestational age at diagnosis, pregnancy outcome, late miscarriage and mode of delivery. Data obtained regarding perinatal outcome were gestational age at birth, birth weight, Apgar score at 5 min, gender, number of days in neonatal care, perinatal survival at 28 days of life, fetal complications including TTTS, sIUGR and congenital anomalies.

Monoamnionicity was diagnosed prenatally with ultrasonography and in some cases confirmed by placental pathology. The first ultrasonography was done in the first or in the second trimester. In Sweden, first trimester ultrasound for all pregnant women was not routine during the study period. If a pregnancy was followed as monoamniotic but later diagnosed as diamniotic by the placental pathology examination, the pregnancy was excluded from analysis. All uncomplicated monoamniotic twin pregnancies are followed as outpatients and are delivered by elective cesarean section in gestational weeks 32–34. In Sweden, respiratory distress syndrome (RDS) prophylaxis with corticosteroids is routine in premature deliveries before gestational week 34 + 0. The timing of RDS-prophylaxis was changed during the study period. Before the first quarter of 2018, RDS prophylaxis was given to premature deliveries before gestational week 33 + 0.

The primary outcomes were live birth rate, neonatal death and perinatal survival. Live birth rate after 22 and 24 weeks gestation and overall live birth rate were presented in this study. Overall live birth rate was defined as the total live birth rate, including spontaneous miscarriage and termination of pregnancy (TOP). Neonatal death was defined as death of the infant before 28 completed postnatal days. Perinatal survival was defined as the survival rate from 22 to 24 weeks gestation to 28 days of life. Secondary outcomes were gestational age at delivery, late miscarriage, and frequency of fetal complications (TTTS, sIUGR and congenital anomalies).

Miscarriage was defined as fetal demise before 22 weeks gestation. Late miscarriage was defined as fetal demise after 12 weeks gestation. To do a proper comparison with international studies, where miscarriage is defined as fetal demise before 24 weeks gestation, live birth rate after 24 weeks was also presented. Definition of the first trimester was < 14 weeks of gestation. Neonatal care days was defined as care at a regular neonatal ward or neonatal intensive care unit, whereas neonatal home care was not included.

### Statistical analyses

Data analyses were performed using IBM SPSS Statistics for Windows, Version 25.0. (Armonk; NY: IBM Corp). Continuous variables were presented as mean ± standard deviation (SD) or median (interquartile range) as appropriate. Categorical variables were presented as absolute values and percentage [*n* (percent)].

### Ethical approval

The study was approved by the Swedish Ethical Review Authority (decision number 2019-00608) the 27th of November 2019.

## Results

Twenty-seven MCMA twin pregnancies (54 fetuses) were identified between 2010 and 2019. There was one case of conjoined twins that was excluded from the study. Another case was followed as MCMA but was postnatally diagnosed as MCDA at the placental pathology examination and, therefore, excluded from the analyses. Three cases were referred from outside the region for fetal therapy and, therefore, excluded. Hence, 22 MCMA twin pregnancies (44 fetuses) were included in the analyses. The prevalence of MCMA deliveries was 1.08% (18/1663) of twin deliveries and 0.02% (18/84935) of all deliveries. Thus, MCMA deliveries represented 11/1000 of twin deliveries and 2/10,000 of all deliveries.

Maternal and pregnancy characteristics are presented in Table 1. Fifteen pregnancies were conceived spontaneously, six by in vitro fertilization (IVF) and one by oocyte donation. The mean maternal age was 32 years (SD ± 5). The mothers had a median parity of one (range 1–3) and a mean BMI of 24 (SD ± 4). Nineteen pregnancies (86%) were diagnosed as monoamniotic in the first trimester. The remaining three (14%) were diagnosed in the second trimester. The mean gestational age at birth was 32.5 weeks (SD ± 1.5).

**Table 1:** Maternal and pregnancy characteristics

Case	Pregnancy type	Age (years)	Parity	BMI	Time of diagnosis (trimester)	Pregnancy outcome	GA at birth (weeks and days)	Emergency/elective cesarean section	Maternal pregnancy complications
1	Spontaneous	28	1	28	1	Miscarriage (14 + 2)			None
2	Spontaneous	34	2	32	1	LB	32 + 2	Elective	None
3	Spontaneous	31	0	22	1	LB	32 + 6	Elective	None
4	Oocyte donation	44	0	22	1	LB	31 + 6	Acute^c^	None
5	Spontaneous	34	3	34	2	LB	33 + 6	Elective	Gestational diabetes
6	IVF	36	1	22	1	LB	30 + 6	Elective^d^	Anemia
7	Spontaneous	30	1	23	1	LB	32 + 3	Elective	Anemia
8	IVF	37	3	23	1	LB	32 + 0	Elective	None
9	Spontaneous	34	2	21	1	LB	33 + 0	Elective	None
10	Spontaneous	36	1	24	1	LB	31 + 2	Elective	None
11	Spontaneous	37	3	25	1	LB	34 + 3	Elective	Thrombocytopenia
12	Spontaneous	24	0	21	1	TOP (15 + 0)^a^			None
13	Spontaneous	27	2	23	2	LB	34 + 3	Elective	None
14	Spontaneous	30	1	18	1	sIUFD (28 + 0)^b^ /LB	33 + 4	Elective	Anemia
15	IVF	34	0	22	1	Miscarriage (13 + 5)			None
16	Spontaneous	31	0	23	1	LB	29 + 1	Acute^e^	None
17	IVF	33	1	22	1	Miscarriage (16 + 2)			None
18	Spontaneous	34	1	28	1	LB	32 + 6	Elective	None
19	Spontaneous	24	0	28	2	LB	29 + 3	Acute^e^	None
20	Spontaneous	33	2	21	1	LB	33 + 5	Elective	None
21	IVF	36	0	21	1	LB	33 + 1	Elective	PPH
22	IVF	27	0	29	1	LB	33 + 4	Elective	Anemia

*GA* gestational age; cases 1, 12, 14 (only twin 2), 15 and 17 were terminated; Miscarriage spontaneous loss of pregnancy before 22 weeks gestation, *LB* live birth, *TOP* termination of pregnancy, *sIUFD* single intrauterine fetal demise, *PPH* postpartum hemorrhage

^a^Terminated due to severe umbilical artery blood flow changes

^b^sIUFD in gestational week 28. At delivery 2 tight true knots of the umbilical cords were observed. No other explanation to sIUFD was found at prenatal ultrasound or postnatal placental pathology examination

^c^Emergency cesarean section due to threatening fetal asphyxia

^d^Elective cesarean section due to sIUGR

^e^Acute cesarean section due to premature rupture of the membrane

Perinatal outcome is presented in Table 2. In 17 of 22 pregnancies (77%), both fetuses were born alive. The live birth rate after 22 weeks gestation was 97%. There were no neonatal deaths. Thus, the perinatal survival rate after 22 and 24 weeks gestation was 97%. The liveborn children had a mean birthweight of 1910 g (SD ± 302 g). Four of the newborn children had a 5 min Apgar score < 7. All children were treated with neonatal care with a mean period of 19 days (SD ± 13 days).

**Table 2:** Perinatal outcome

Case	Birthweight (g) t1	Birthweight (g) t2	Gender	Apg5 twin 1	Apg5 twin 2	Neonatal care (days)^b^	PNS (*n* fetuses)
1	Miscarriage						
2	2180	2194	Male	8	9	13^a^	2
3	2140	2020	Female	9	9	12^a^	2
4	2011	1850	Male	5	9	46^a^	2
5	2140	2545	Female	9	8	10	2
6	1470	1230	Female	8	9	35^a^	2
7	1710	1890	Female	5	7	20^a^	2
8	1925	1765	Male	9	9	16	2
9	2163	2003	Female	9	8	12	2
10	1960	1894	Female	7	5	26^a^	2
11	2198	1914	Female	10	10	9	2
12	TOP						
13	2082	2017	Male	10	10	6	2
14	2290	sIUFD	Female	7	sIUFD	8	1
15	Miscarriage						
16	1238	1404	Female	8	10	41	2
17	Miscarriage						
18	1879	2005	Male	7	5	18	2
19	1384	1457	Female	9	9	40	2
20	1925	1860	Female	6	9	12^a^	2
21	1675	1714	Female	9	8	12	2
22	2042	2160	Male	10	10	7	2

*t1* twin 1, *t2* twin2, *Apg5* Apgar score after 5 min, Miscarriage, spontaneous loss of pregnancy before 22 weeks gestation, *TOP* termination of pregnancy, *PNS* perinatal survival

^a^Due to RDS

^b^Neonatal home care not included

Three of 22 pregnancies ended in late miscarriage (14%). There was one pregnancy (case 5) with mild TTTS. This was detected at 25 weeks of gestation and treated with amniodrainage only. TTTS resolved following the procedure. There were no cases of sIUGR. Two of the infants (twin 2, case 6 and twin 2, case 20) were born small for gestational age. There was one case of unexpected sIUFD and no case with dIUFD.

Five of 44 fetuses (11%) from four pregnancies were diagnosed with congenital anomalies. In one case both children were born with anomalies affecting one of the thumbs. One of the children in this pregnancy also had a cardiac ventricular septal defect. One child was born with a hemivertebrae and butterfly vertebrae. In another case one of the fetuses had CNS malformations that were detected prenatally. This pregnancy ended in spontaneous miscarriage before a planned termination of the affected fetus. In another case, one of the children was born with situs inversus. All congenital anomalies, except one, were detected postnatally.

All children were treated for mild or moderate prematurity related complications, but there were no severe neonatal complications. Fourteen children were treated for infant Respiratory Distress Syndrome (RDS); however, all resolved with treatment and no child developed bronchopulmonary dysplasia.

## Discussion

Perinatal survival in MCMA twin pregnancies managed at Karolinska University Hospital was high and the frequency of fetal and neonatal complications was low. The live birth rate of 97% after 22 and 24 weeks of gestation in our cohort is comparable with the results above 90% reported by studies with similar sample sizes and criteria. One study including 25 MCMA pregnancies, presented a live birth rate of 98% after 20 weeks gestation (congenital anomalies included) [11]. Another study with a larger sample size presented live birth rate of 92% after 26 weeks gestation (congenital anomalies excluded) [4]. A recently published systematic review and meta-analysis by D’Antonio et al*.* presented a live birth rate of 94% after 24 weeks gestation [5].

Studies with larger sample sizes have demonstrated overall live birth rates between 82 and 84% [3, 12]. This is in concordance with an overall live birth rate of 80% in our study (congenital anomalies included).

A multicenter study including 61 MCMA pregnancies was recently performed in Denmark by Madsen et al*.* [13]. In Denmark the management of MCMA pregnancies is similar to the management in Sweden. In this study MCMA deliveries represented 1/10,000 of all deliveries and 5/1000 of twin deliveries. In our cohort, the rate of MCMA deliveries represented 2/10,000 of all deliveries and 11/1000 of twin deliveries. A possible explanation to the higher rate in our study is that a majority of MCMA pregnancies, as complicated twin pregnancies, is referred to our hospital. The overall live birth rate in the Danish study was 56%, which is lower than our result of 80% (congenital anomalies included). In Denmark, all pregnant women are offered a first trimester scan at 11–13 weeks of gestation [13]. In Sweden, the routine anomaly scan is performed in the second trimester and not all pregnant women are scanned before this. Therefore, it is possible that cases of MCMA pregnancies that were spontaneously miscarried before detection were not included in our cohort. After 22 weeks the live birth rates were 88% compared to the results of the present study of 97% (congenital anomalies included). A possible explanation of the higher live birth rate of MCMA pregnancies in our study, might be the different study design and sample sizes of the two studies and that all our cases were managed in a tertiary center. The Danish study was population based and a multicenter study.

The frequency of late miscarriages in the current study was 14%. This is similar to the results presented by two studies that have reported frequencies of 16 and 17% [3, 14]. Other studies have reported higher frequencies of late miscarriages, with rates of 26–27% [2, 13]. With a rate of congenital anomalies of 11%, TTTS of 5% and no cases of sIUGR the frequency of complications in our study was quite low (TOP included). However, the interpretation of results is limited by the small sample size.

The prevalence of congenital anomalies in MCMA pregnancies has earlier been reported as 18–28% [8]. The prevalence of congenital anomalies is probably underestimated in many studies. It is possible that some MCMA pregnancies complicated by congenital anomalies are spontaneously miscarried before the anomalies are detected. Furthermore, congenital anomalies in one fetus is a common reason for selective termination. Therefore, exclusion of terminated pregnancies or pregnancies that underwent reduction could result in a deceptively low frequency of congenital anomalies. In a recent systematic review and meta-analysis 5% (44/888) of MCMA twin pregnancies were affected by TTTS [15]. The higher prevalence of arterioarterial anastomoses in MCMA-placentas, which are protective against the pathology in TTTS, are the probable explanation of the lower incidence in MCMA pregnancies [16, 17]. It could be that cases of TTTS in MCMA are not detected as often as in MCDA pregnancies, since the fetuses share the same amniotic sac. The differences in amniotic fluid, used as a diagnostic criterion in MCDA [9], cannot be used when diagnosing TTTS in MCMA. Thus, it is possible that MCMA cases with TTTS remain undetected. It is not unlikely that undiagnosed TTTS cases constitute part of the pregnancies that are lost in IUFD before 24 weeks gestation.

Seven of 22 pregnant women had maternal complications due to twin pregnancy. The complications were anemia, gestational diabetes, thrombocytopenia and postpartum hemorrhage. However, many women probably delivered before the onset of possible pregnancy complications, such as preeclampsia, pregnancy-induced hypertension or intrahepatic cholestasis, as these were not seen in this material.

Saccone et al. [4] have studied the outcome of MCMA pregnancies managed as inpatients versus outpatients. The results suggested that perinatal mortality rate was lower in the inpatient group, but the results were inconclusive. However, this has been contradicted by others [18]. The hemodynamic instability between the two fetuses is a causal factor behind sudden fetal demise in these pregnancies. These are probably instantaneous changes which are very difficult to anticipate, even with inpatient management. Moreover, when discussing in-versus outpatient management of these pregnancies, the possible advantages with inpatient management have to be weighed against disadvantages, such as psychological consequences for the mother [8].

When planning delivery of MCMA pregnancies the risk of neonatal complications has to be weighed against the risk of IUFD. In a recently published systematic review and meta-analysis by Buca et al., it is suggested that the risk of composite neonatal morbidity is high in uncomplicated MCMA pregnancies. This risk gradually decreases with an increased gestational age at delivery and between 33 and 34 weeks there is a substantial reduction in neonatal morbidity [19]. According to van Mieghem et al., the cutoff point between the risk of non-respiratory neonatal complications and the risk of IUFD was at gestational age 32 + 4 [12]. Based on these results, delivery between gestational weeks 32 and 34 is recommended [9, 12]. The mean gestational age at delivery in our cohort was 32.5 weeks. There was only one case of fetal demise after 22 weeks gestation, which occurred at gestational week 28. In a recently published meta-analysis, the highest rate of IUFD occurred before 30 weeks gestation [5]. In another study, no fetal deaths occurred after 32 weeks gestation [4]. There were 134 children delivered after 33 weeks gestation and 46 children delivered after 34 weeks gestation in that study [4]. Our results in combination with the results presented by these studies support that delivery of MCMA pregnancies could wait until gestational week 33 + 0 when possible.

To improve perinatal outcome in these rare pregnancies, it might be preferable to centralize the regional management of MCMA pregnancies. Theoretically, this might increase timely identification of complications and possibly improve perinatal outcome.

The major strength of this study is that it presents the largest data on MCMA pregnancies in Sweden, including all consecutive cases in a tertiary center. Main limitations are the small number of cases and the retrospective design which adds a risk of bias. Another limitation is that not all MCMA twins were diagnosed in the first trimester of pregnancy. Despite these limitations, it is important to increase knowledge of outcome in these rare pregnancies, to be able to properly counsel the patients.

## Conclusions

The perinatal survival of MCMA pregnancies managed at Karolinska University Hospital was high (97%) and comparable to previously published studies. Mean gestational age at birth was 32.5 weeks and fetal and neonatal complication rates in MCMA pregnancies were low.

## Data Availability

Not applicable.

## Abbreviations

dIUFD: Double intrauterine fetal demise
IUFD: Intrauterine fetal demise
IUGR: Intrauterine growth restriction
MCDA: Monochorionic diamniotic
MCMA: Monochorionic monoamniotic
RDS: Respiratory distress syndrome
sIUFD: Single intrauterine fetal demise
TTTS: Twin–twin transfusion syndrome

## Code availability

Data analyses were performed using IBM SPSS Statistics for Windows, Version 25.0. (Armonk; NY: IBM Corp).
